# Reconstruction of lateral knee joint stability following resection of proximal fibula tumors

**DOI:** 10.3892/etm.2013.1429

**Published:** 2013-11-26

**Authors:** SHI-CHANG ZHAO, CHANG-QING ZHANG, CHUN-LIN ZHANG

**Affiliations:** 1 Department of Orthopaedics, Shanghai Sixth People’s Hospital, Shanghai Jiaotong University, Shanghai 200233, P.R. China; 2 Department of Orthopaedics, Shanghai Tenth People’s Hospital, Tongji University, Shanghai 200072, P.R. China

**Keywords:** proximal fibula tumor, functional reconstruction, knee stability, Musculoskeletal Tumor Society score

## Abstract

Managing tumors of the proximal fibula may require en bloc resection of the fibular head with the attachment site of the lateral collateral ligament (LCL) and biceps femoris tendon. The aim of the present study was to evaluate knee stability and the Musculoskeletal Tumor Society (MSTS) functional score of patients with proximal fibula tumors. Twenty-nine patients with proximal fibula tumors were retrospectively reviewed (18 patients in the reconstruction group and 11 patients in the non-reconstruction group). A comparative analysis was conducted of knee stability (measuring the degree of lateral joint space opening using varus stress radiographs with a 30º knee flexion) and MSTS functional score between the two groups. The mean follow-up period was 42.8±20.9 months (range 24–117) and 40.8±26.0 months (range 24–117) for the reconstruction and the non-reconstruction groups, respectively. Fifteen patients (83.3%) in the reconstruction group had a stable knee, one (5.6%) had grade 1 instability and two (11.1%) had grade 2 instability. Four patients (36.4%) in the non-reconstruction group had a stable knee, three (27.3%) had grade 1 instability, one (9.1%) had grade 2 instability and three (27.3%) had grade 3 instability. Patients who underwent reconstructive surgery exhibited a higher rate of knee stability compared with those in the non-reconstruction group (P<0.05). The MSTS function scores were 93% (range, 93–100%) for the reconstruction group and 87% (range, 60–100%) for the non-reconstruction group (P<0.05). Reconstruction of the LCL and biceps femoris tendon to the lateral tibial metaphysis with a suture anchor was a safe, reliable and simple technique following resection of proximal fibula tumors.

## Introduction

The fibula is a rare anatomical location for malignant primary bone sarcomas and metastatic lesions (1). The proximal fibula is the most common area of the fibula to be affected by tumors; and osteosarcoma, giant cell tumors, chondrosarcoma and aneurysmal bone cysts are the most common type of tumor to develop at this location. The proximal fibula osteosarcoma in the Mayo series reported an incidence of 2% (2). Resection of an aggressive or malignant tumor of the proximal fibula necessitates an en bloc extra-articular resection of the proximal fibula [the proximal tibiofibular joint (PTFJ) transmits loads between the knee and ankle during weight bearing], as well as the lateral collateral ligament (LCL) and biceps femoris tendon, which attach to the proximal fibula, leading to varying degrees of knee instability (3,4). Studies have revealed that the LCL is a predominant constraint to primary varus rotation at all positions of knee flexion (5). Isolated sectioning of the LCL resulted in a marginal but significant increase in varus rotation at all angles of knee flexion.

The biceps femoris imparts a posteriorly directed force to the proximal tibia and the iliotibial band, leading to anterior stability, thus reducing strain on the anterior cruciate ligament (6). The method of lateral-knee reconstruction to improve stability and whether conducting an early reconstruction leads to an improved functional outcome, remains controversial (4,7–9).

Therefore, the aim of the present study was to evaluate the use of suture anchors (used to attach the LCL and biceps femoris tendon to the lateral tibial metaphysis) and compare the postoperative lateral knee stability and functional outcomes with those of patients that had not received reconstruction.

## Subjects and methods

### Patients

Between January 2006 and December 2009, 29 proximal fibula tumor resections were performed. Eighteen of these resections involved reconstruction of the LCL and biceps femoris tendon to the lateral tibial metaphysis using suture anchors. A further 11 proximal fibula tumor resections without surgical reconstruction served as the non-reconstruction group. All tumors were histopathologically defined from biopsied specimens and the histological diagnoses are presented in Table I. This study was conducted in accordance with the declaration of Helsinki. Approval was obtained from the Ethics Committee and the Institutional Review Board of Shanghai Sixth People’s Hospital, Shanghai Jiaotong University (Shanghai, China). Written informed consent was obtained from all participants.

### Preoperative preparation

Preoperative detailed history, a comprehensive physical examination and adequate imaging studies, including X-ray, computed tomography, magnetic resonance imaging, single photon emission computerized tomography and magnetic resonance angiography, were required. The imaging studies assisted in defining tumor staging, the extent of bone destruction, intramedullary involvement and soft-tissue extension. In addition, the position of the tumor to the nerves, blood vessels and tibia was noted. Biopsies were performed with an anterolateral approach in the safe area formed by the fibular head and the deep peroneal nerve in the anterior compartment. This was required to protect the peroneal nerves and LCL from contamination by tumor tissue during biopsy. This applied to benign lesions and to malignant bone tumors (10).

### Type of proximal fibula resection

**Figure 1:**
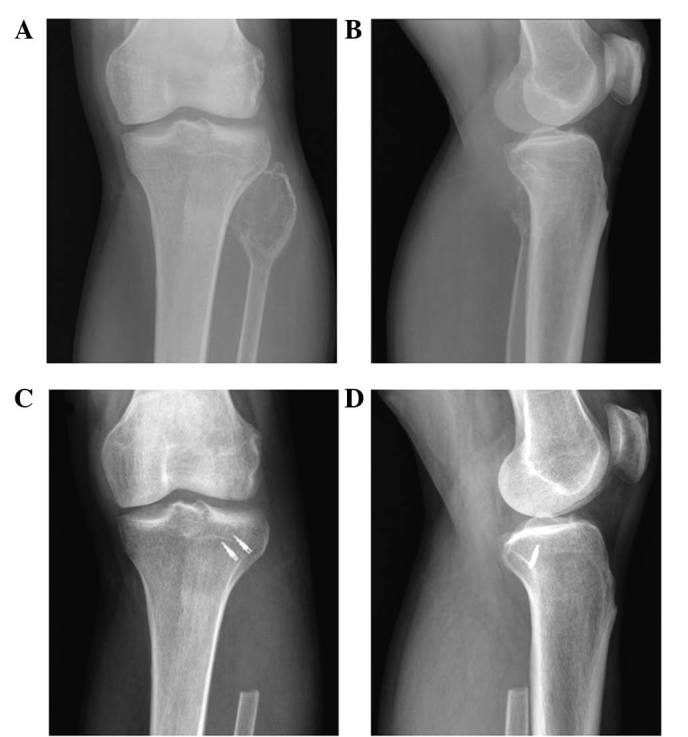
(A and B) Preoperative plain radiograph shows a 35-year-old male with a giant cell tumor of the proximal fibula. (C and D) Postoperative plain radiograph shows the lateral collateral ligament and biceps femoris tendon reattached to the lateral tibial metaphysis.

The types of proximal fibula resection have been previously described by Malawer (11). A type I proximal fibula resection is reserved for benign aggressive, low-grade malignant tumors and metastatic tumors (Fig. 1). It includes intra-articular resection of the proximal fibula with 2–3 cm of normal diaphysis, with a thin muscle cuff in all dimensions and the LCL attachment site. The anterior tibial artery is occasionally sacrificed. The peroneal nerve and its motor branches may be preserved. A type II resection is reserved for high-grade malignant tumors, which usually have considerable cortical destruction with extra-osseous extension. This resection includes an extra-articular resection of the proximal fibula with 6 cm of normal diaphysis, the anterior and lateral muscle compartments, the anterior tibial artery and occasionally, the peroneal artery and peroneal nerve.

### Surgical management

A semisupine position (45º elevation of the operated side) was used to permit easy access to the anterior and lateral compartments and allow dissection of the popliteal space. A single utilitarian approach provided safe and wide exposure of all four compartments of the leg and popliteal fossa, thus allowing the resection of proximal fibula tumors. The incision began posteriorly, ~8 cm proximal to the midpoint of the transverse popliteal skin crease, then curved gently forward and distally toward the anterior tibial crest, finally passing anteriorly to the fibular head and over Gerdy’s tubercule to a point just lateral of the tibial crest. The incision extended 5 cm distally to the level of the planned osteotomy. When a primary bone sarcoma was resected, the previous biopsy tract, with a 2–3 cm margin, was included in the incision. A large lateral flap and a smaller medial flap was developed.

**Figure 2:**
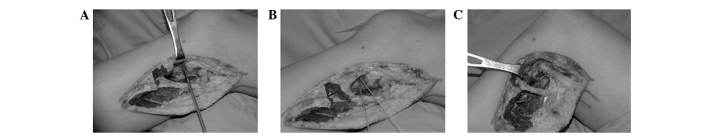
The lateral collateral ligament and biceps femoris tendon are reattached to the lateral tibial metaphysis following type I en bloc resection using suture anchors with a 20º knee flexion.

Five consecutive steps were performed in the type II resection: i) Exploration of the common peroneal nerve, which encompasses the base of the fibular head, to enter the peroneus longus tunnel; ii) exploration of the popliteal space and blood vessels. Large tumors of the proximal fibula may reach the midline posteriorly and push on the popliteal vessels. The predominant vessels were exposed by reflecting the lateral gastrocnemius muscle through its length and, if required, released the proximal tendinous origin from the lateral femoral condyle; iii) excision of the anterior and lateral muscle compartments. The anterior and lateral musculature and the overlying deep fascia were excised. The distal level of transection was at the musculotendinous junction. The LCL and the biceps femoris tendon were released 2.5 cm proximally to their fibular insertion to prepare for subsequent reconstruction; iv) extra-articular resection of the PTFJ. A semicircular incision was made directly through the popliteus muscle towards the posterior aspect of the lateral tibial condyle; including the proximal fibula, part of the lateral condyle of the tibia and the PTFJ. Following osteotomy, it was important to inspect the lateral tibial condyle. If the knee joint capsule was exposed and opened, it was repaired in order to prevent a potential synovial fistula; iv) soft-tissue reconstruction. The LCL and biceps femoris tendon were attached to the lateral tibial metaphysic using 5.0-mm suture anchors (DePuy Mitek Inc., Raynham, MA, USA) with a 20º knee flexion (0.3 cm below the PTFJ, a Bunnell braided suture was recommended to reinforce the fixation with nonabsorbable sutures to the overlying iliotibial band and fascia) (Fig. 2). The exposed tibia and soft-tissue defect was closed and covered. It was possible to rotate the lateral gastrocnemius muscle to cover the defect when the muscle was released close to its origin through the muscle substance and from its tendinous insertion at the distal end. Care was taken to preserve its proximal pedicle, the lateral sural artery, throughout the dissection.

### Postoperative management

Suction drainage and prophylactic antibiotics were continued for 3–5 days. The leg was kept elevated during this time. The extremity was immobilized at a 20º knee flexion for 2–3 weeks to allow soft-tissue healing and posterior capsule reattachment. Full weight-bearing was allowed when the limb had been immobilized in a cast. An ankle-foot orthosis was required following a type II resection unless tenodesis of the anterolateral compartment to the tibial shaft had been performed. Patients with high-grade sarcomas were treated with postoperative chemotherapy. Patients with Ewing’s sarcoma were further treated with radiation therapy consisting of external beam radiation of 6,000–7,000 cGy.

### Data analysis

Data with regard to histological diagnoses, surgical techniques of tumor resection and reconstruction, complications, knee stability and Musculoskeletal Tumor Society (MSTS) functional score were recorded. Patients were evaluated by plain radiography and a physical examination every three months for the first two postoperative years.

Lateral knee stability was assessed by measuring the degree of lateral joint space opening using valgus-varus stress radiographs with a 30º knee flexion and in neutral tibial rotation. Instability was scored as grades 1–3: grade 1, an opening of 1–5 mm; grade 2, 6–10 mm; and grade 3, ≥11 mm (e.g. complete LCL dysfunction). Grade was determined by comparing the results with that of a normal contralateral knee (12).

Functional evaluation was conducted according to the MSTS functional scoring system (13). With this system, each answer was scored based on frequency of symptoms using a five-point scale between zero (indicating a significant problem) and five (indicating no problem or full, normal function). Responses to all questions were combined for a composite score ranging between 0 and 30, with higher scores indicating improved knee function. The results are expressed as the proportion of full normal function in all six categories (pain, function, emotional acceptance, supports, walking and gait) and are based on the values recorded during each patient’s most recent follow-up.

### Statistical analysis

Data were analyzed using the SPSS 16.0 software package (SPSS Inc., Chicago, IL, USA). Comparison of functional parameters and knee stability following surgery was performed using the Wilcoxon Two-Sample test. P<0.05 was considered to indicate a statistically significant difference.

## Results

The mean follow-up period was 42.8±20.9 months (range 24–117) for the reconstruction group and 40.8±26.0 months (range 24–117) for the non-reconstruction group. The reconstruction group consisted of 11 males and 7 females, with an average age of 31.5±13.5 years (range, 18–45 years). The non-reconstruction group consisted of 6 males and 5 females, aged 32.5±14.5 years (range, 18–47 years).

No complications were identified in association with the surgical excision, including skin necrosis, infection, hematoma or thrombophlebitis and synovial fistula. All 7 patients (3 in the reconstruction group and 4 in the non-reconstruction group) who received type II resections had an expected iatrogenic permanent loss of peroneal nerve function. Three patients (2 in the reconstruction group and 1 in the non-reconstruction group) who received type I resections had a transient peroneal nerve palsy that resolved spontaneously within three to six months.

Fifteen patients (83.3%) in the reconstruction group had stable knee (12 with type I resection, 3 with type II resection), 1 (5.6%) had grade 1 instability and 2 (11.1%) had grade 2 instability (Table II). The patient with grade 1 instability was asymptomatic and did not require knee support for ambulation. Four of the 11 patients (36.4%) in the control group had a stable knee, 3 (27.3%) patients had grade 1 instability, 1 (9.1%) had grade 2 instability and 2 (27.3%) had grade 3 instability (Table II). The patients with grade 2 and 3 instability in the control group required a knee brace and occasionally, a cane for ambulation. Overall, patients in the reconstruction group had a higher rate of knee stability than those in the control group (P=0.0099). Furthermore, the results revealed that for type I resections, the reconstruction group had a higher rate of knee stability than the non-reconstruction group (P=0.0165), while for type II resections, no statistical difference was observed (P=0.0615).

**Figure 3:**
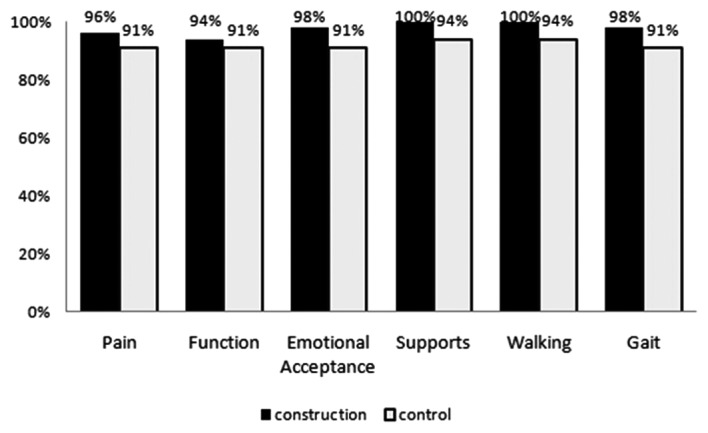
Functional outcomes for patients in the reconstruction group with type I and II fibular resection.

**Figure 4:**
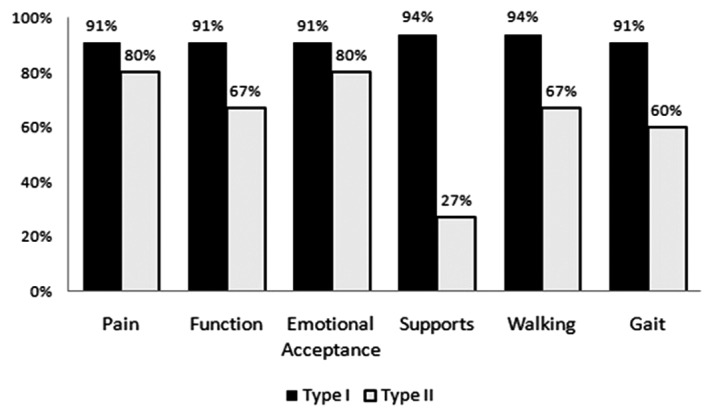
Functional outcomes for patients in the non-reconstruction group with type I and II fibular resection.

**Figure 5:**
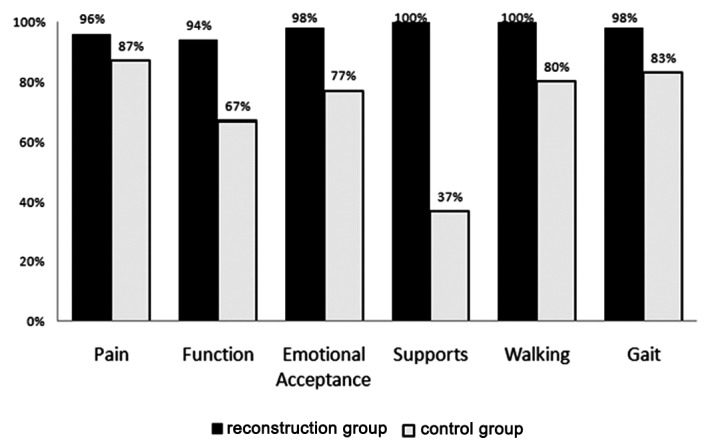
Musculoskeletal Tumor Society function scores restricted to the resection type, with results showing type I resection, patients in the reconstruction group had higher function scores than those in the non-reconstruction group.

**Figure 6:**
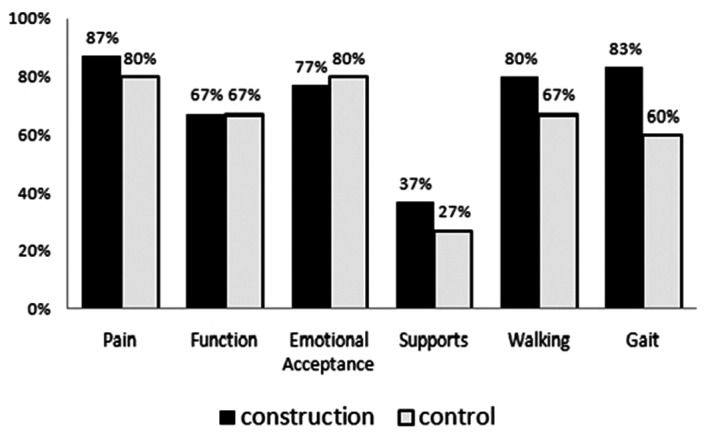
Among those that received type II resection, patients who received reconstruction scored higher (improved knee function) than those without, in all categories of Musculoskeletal Tumor Society function scores, with the exceptions of function and emotional acceptance. The reconstruction group had higher composite scores than those in the non-reconstruction group.

MSTS function scores were available for 16 patients in the reconstruction group and 10 patients in the non-reconstruction group. There was 1 fatality in the reconstruction group and 2 in the non-reconstruction group due to metastases (bone and lung). In the reconstruction group, the composite scores ranged between 93 and 100%, with a median of 93%. In the non-reconstruction group, the composite scores ranged between 60 and 100%, with a median of 87%. In general, patients in the reconstruction group had higher composite MSTS function scores than those in the non-reconstruction group (P<0.05). For the reconstruction and non-reconstruction groups, in all categories of MSTS, patients who had received type I resection scored higher (improved knee function) than those who had received type II resection (Figs. 3 and 4). By analyzing MSTS function scores restricted to those who received type I resection, results suggested that in all categories of MSTS, those who received reconstruction scored higher than those without reconstruction (P<0.05) (Fig. 5). Similarly, among those that received type II resection, patients who received reconstruction scored higher than those without in all categories of MSTS, with the exception of function and emotional acceptance; the reconstruction group had higher composite scores than those in the non-reconstruction group (P<0.05) (Fig. 6).

In the reconstruction and non-reconstruction groups, patients with type I resection had a higher rate of knee stability (P<0.01 and P<0.05, respectively) and an improved functional outcome (P<0.001 and P<0.05, respectively) than those with type II resection.

## Discussion

Tumors of the proximal fibula are rare. Patients with locally aggressive tumors require surgical management and various extensile approaches of the fibula and popliteal vessels have been described for limb-salvage procedures (14,15). The proximal fibula, serving as the point of insertion of the LCL and biceps femoris, is integral in the lateral stabilization of the knee. Therefore excision of the proximal fibula may disrupt lateral stability. It is essential to repair the LCL and biceps femoris following proximal fibular excision (11). The present study communicates our reconstruction technique and analyzed lateral knee stability and functional outcome following resection the proximal fibula. This technique included fixation of the LCL and biceps femoris tendon to the tibial metaphysis, immobilization and protected weight-bearing.

Siddiqui *et al*(16) demonstrated that, following resection of the proximal fibula chondroblastic osteosarcoma, the knee function remained stable, although there was no attempt to reconstruct the lateral soft tissue structures.

Kanazawa *et al*(17) demonstrated promising results in three stage IIB proximal fibula osteosarcomas by preserving the common peroneal nerve through intentional marginal excision without surgical reconstruction.

Einoder and Choong (8) reported that the knee remains functionally stable following resection of proximal fibula tumors, without reconstruction of the LCL for four years of follow-up. However, a 1 cm joint space widening was detected in two cases (which may result in osteoarthritis in long term follow-up).

Takahashi *et al*(9) observed 13 osteosarcomas of the proximal fibula. The LCL and biceps femoris tendon was reattached to the lateral wall of the tibia with a staple in two cases, with a suture anchor in one case and with simple sutures to the soft tissues in six cases. No patient presented with knee instability or exhibited valgus instability on physical examination. It was therefore indicated that surgical reconstruction of the LCL was not required to achieve optimal function. This may be due to the sparing of other stabilizing structures, including the cruciate ligaments.

Draganich *et al*(3) reported six patients with proximal fibular resection where repair of the LCL and biceps femoris was performed. Gait and knee stability was evaluated with an instrumented system and increased anterior and anteroposterior translation of the knee during flexion, varus-valgus rotations at 20º flexion and several abnormalities in ground reaction forces were identified. It was concluded that proximal fibular resection without ligamentous reattachment resulted in gait abnormalities and knee instability, and it is possible to minimize these disorders by proper reattachment of the LCL and biceps tendon at their novel insertion site.

Abdel *et al*(18,19) reported that in 112 malignant and 121 benign proximal fibula tumors, no long-term knee instability was observed in the patients who underwent resection with the LCL and biceps femoris tendon reconstruction by staple or suture anchor.

Faezypour *et al*(20) described a similar technique of reconstructing the LCL and biceps femoris tendon; however, the 5 patients in this study had benign tumors and underwent type I resections of the proximal fibula.

Saini *et al*(21) communicated observations following Malawer type II resection in 8 patients with proximal fibular osteosarcomas. Following tumor resection, nonabsorbable sutures (no. 5 Ethibond; Ethicon Endo-Surgery (Europe) GmbH, Norderstedt, Germany) were used to reattach the stumps of the LCL and biceps femoris through drill holes in the lateral wall of the proximal tibia. The LCL and biceps femoris were reinforced by suturing them to the overlying iliotibial band. According to follow-up varus stress radiographs, two patients had stable knees, 5 had grade 1 laxity and 1 had grade 2 laxity.

In the present study a group of patients who underwent two types of resections for benign (type I resection) and malignant (type II resection) diagnoses were analyzed and the stability and functional outcomes between the reconstruction group and the control group were compared. This is a relatively small series (and therefore may limit the statistical power of the data); however, it was possible to identify differences in lateral knee joint stability and functional outcome with the two types of resection.

Consideration of the resection type (I vs. II) may be a significant factor contributing to knee stability following surgery; therefore knee stability was compared by surgical type between the reconstruction and non-reconstruction groups. Knee stability was assessed by the surgeons who were aware of the type of fibular resection performed on the patients at the time of assessment. Generally, patients with type I resection had a higher rate of knee stability than those with type II resection. For type I resections, it was observed that the reconstruction group had a higher rate of knee stability than the non-reconstruction group, while for type II resections, no statistical difference was revealed.

The patients who received type II resection of the fibula had greater lateral knee instability and lower functional outcome scores, requiring an orthotic device and sometimes additional surgery, such as tenodesis of the toe extensors and the anterior tibial muscle. The majority of patients who received type I resection exhibited a stable knee or a mild grade 1 instability that was asymptomatic and did not require knee support. We hypothesize that the reason for the increased instability after a type II resection may be the shorter LCL and biceps femoris tendon stumps, which provide a short lever arm for knee function and less viable adjacent soft tissue to support healing. Furthermore, delayed healing is anticipated in patients receiving postoperative chemotherapy. In addition to the impairment of the LCL and biceps femoris tendon function, patients who have a type II resection lose the peroneal nerve and a considerable quantity of muscle tissue from the anterolateral compartment of the leg. These losses are considered to be the reason for their inferior functional outcome when compared with outcomes of patients who received a type I resection. Of all functional parameters assessed, the most profound difference between the two groups was the requirement for supports due to the use of peroneal braces in patients who received a type II resection.

When comparing knee stability between the reconstruction and control groups following type II resection, no statistical differences were identified, this was not the expected outcome. This may be due to the small sample size of patients with type II resections in the reconstruction (n=3) and non-reconstruction (n=4) groups, limiting the statistical power of the data. Further reasons for the lack of a statistical difference in knee stability between the reconstruction and non-reconstruction groups, following type II resection, may be associated with the lateral knee joint stability structures, which have been resected to a greater degree. The LCL and biceps femoris tendon stumps are subsequently shorter, rendering reconstruction difficult and less effective as there is less, viable adjacent soft tissue to support its healing. In type II resection, a gastrocnemius flap is used for soft tissue reconstruction. This procedure alone results in additional mechanical problems. Kramers *et al* noted that following a gastrocnemius flap procedure, the knee develops a compensatory mechanism during the swing phase of the gait by increasing peak knee flexion; however, knee motion remains normal in the stance phase (22).

LCL may provide the main resistance to varus rotation at the knee, whereas the biceps femoris may be a significant dynamic restraint to anterior displacement of the tibia (23). In conclusion, the reconstruction of the LCL and biceps femoris tendon to the lateral tibial metaphysis, with suture anchors, was observed to be a safe and reliable technique to reconstruct knee stability following resection of the proximal fibula. It provided stability and optimal function in the majority of patients. This technique is simple to perform and associated with minimal levels of morbidity. However, a multicenter study and a greater number of long-term follow-up dates is required to demonstrate whether this method of reconstruction delays the occurrence of knee osteoarthritis.

## Figures and Tables

**Table I:** Histological diagnoses of included subjects.

Histological diagnosis	Reconstruction group (n=18)	Non-reconstruction group (n=11)
Giant cell tumor	7	4
Aneurysmal bone cyst	5	2
Chondrosarcoma	1	1
Osteosarcoma	2	2
Ewing’s sarcoma	0	1
Benign fibrous histiocytoma	3	1

**Table II:** Knee stability in the reconstruction and non-reconstruction groups.

	Reconstruction group (n=18)		Non-reconstruction group (n=11)	
		
Outcome	Type I	Type II	Type I	Type II
Stable knee	12	3	4	0
Lateral knee instability				
Grade 1	0	1	2	1
Grade 2	0	2	0	1
Grade 3	0	0	1	2
